# Supplementary material for: Histamine Metabolism in IBD: Towards Precision Nutrition
Source: Nutrients. 2025 Jul 29;17(15):2473. doi: 10.3390/nu17152473 (PMC12348536; doi:10.3390/nu17152473)
Supplement: Supplementary file 1 [file nutrients-17-02473-s001.zip › nutrients-3754994-supplementary.pdf]

Supplementary Table S1

Histamine Metabolism Part
("Inflammatory Bowel Diseases"[MeSH] OR "Crohn Disease"[MeSH] OR "Ulcerative Colitis"[MeSH] OR "inflammatory bowel disease" OR "Crohn's disease" OR "ulcerative colitis")
AND
("Histamine"[MeSH] OR "histamine")
AND
("immune system" OR "diamine oxidase" OR "histamine intolerance" OR "biogenic amines" OR "mast cell" OR "tryptase" OR "chymase" OR "histamine 2 receptor" OR "H2R" OR "histamine 4 receptor" OR "H4R" OR "chronic inflammatory disease")("Inflammatory Bowel Diseases"[MeSH] OR "Crohn Disease"[MeSH] OR "Ulcerative Colitis"[MeSH] OR "inflammatory bowel disease" OR "Crohn's disease" OR "ulcerative colitis")
**Precision Nutrition/Low-Histamine Diet Part**
("Inflammatory Bowel Diseases"[MeSH] OR "Crohn Disease"[MeSH] OR "Ulcerative Colitis"[MeSH] OR "inflammatory bowel disease" OR "Crohn's disease" OR "ulcerative colitis")
AND
("Low histamine diet" OR "Mediterranean diet" OR "Specific Carbohydrate Diet" OR "Low FODMAP diet" OR "precise nutrition" OR "personalised nutrition" OR "personalized nutrition" OR "Probiotics"[MeSH] OR "probiotics" OR "Prebiotics"[MeSH] OR "prebiotics" OR "supplements" OR "fibre" OR "fiber" OR "gluten-free diet" OR "plant-based diet" OR "Exclusive enteral nutrition" OR "partial enteral nutrition" OR "elimination diet" OR "New Nordic Diet" OR "healthy diet")
